# Describing the monthly variability of hospital-onset *Clostridioides difficile* during early coronavirus disease 2019 (COVID-19) using electronic health record data

**DOI:** 10.1017/ice.2023.171

**Published:** 2024-03

**Authors:** Kaiting Lang, T. J. Atchison, Priti Singh, David M. Kline, James B. Odei, Jennifer L. Martin, Justin F. Smyer, Shandra R. Day, Courtney L. Hebert

**Affiliations:** 1 Division of Epidemiology, College of Public Health, Columbus, Ohio; 2 College of Medicine, The Ohio State University, Columbus, Ohio; 3 Department of Biomedical Informatics, College of Medicine, The Ohio State University, Columbus, Ohio; 4 Department of Biostatistics and Data Science, Wake Forest University, School of Medicine, Winston-Salem, North Carolina; 5 Division of Biostatistics, College of Public Health, The Ohio State University, Columbus, Ohio; 6 Department of Clinical Epidemiology, The Ohio State University Wexner Medical Center, Columbus, Ohio; 7 Division of Infectious Diseases, The Ohio State University Wexner Medical Center, Columbus, Ohio

## Abstract

**Objective:**

To assess the relative risk of hospital-onset *Clostridioides difficile* (HO-CDI) during each month of the early coronavirus disease 2019 (COVID-19) pandemic and to compare it with historical expectation based on patient characteristics.

**Design:**

This study used a retrospective cohort design. We collected secondary data from the institution’s electronic health record (EHR).

**Setting:**

The Ohio State University Wexner Medical Center, Ohio, a large tertiary healthcare system in the Midwest.

**Patients or participants:**

All adult patients admitted to the inpatient setting between January 2018 and May 2021 were eligible for the study. Prisoners, children, individuals presenting with *Clostridioides difficile* on admission, and patients with <4 days of inpatient stay were excluded from the study.

**Results:**

After controlling for patient characteristics, the observed numbers of HO-CDI cases were not significantly different than expected. However, during 3 months of the pandemic period, the observed numbers of cases were significantly different from what would be expected based on patient characteristics. Of these 3 months, 2 months had more cases than expected and 1 month had fewer.

**Conclusions:**

Variations in HO-CDI incidence seemed to trend with COVID-19 incidence but were not fully explained by our case mix. Other factors contributing to the variability in HO-CDI incidence beyond listed patient characteristics need to be explored.

Coronavirus disease 2019 (COVID-19) had a significant impact on hospitalizations over the last 2 years. Not surprisingly, the CDC reports that healthcare-associated infections were much more common in 2020 than in 2019, with an increase in ventilator-associated pneumonia of 45% and an increase in catheter-associated bloodstream infections of 47%.^1^ However, there were significant decreases in hospital-onset *Clostridioides difficile* infection (HO-CDI) rates compared to 2019.^1^ This finding is interesting given the complex factors associated with *C. difficile* transmission and the impact that the COVID-19 pandemic had on these factors.

The major risk factors for HO-CDI include antibiotic use, increasing age, environmental contamination, and comorbidities such as inflammatory bowel disease, kidney disease, and immunodeficiency.^2,3^ Early in the pandemic, one analysis reported that 72% of patients received antimicrobial therapy while hospitalized for COVID-19.^4^ The increased use of antibiotics during the COVID-19 pandemic could have led to a comparative increase in rates of CDI in hospitalized patients. At the same time, there was also increased emphasis on personal protective equipment, hand hygiene, and social distancing in hospital settings and elsewhere during the pandemic, which may have reduced inpatient transmission of *C. difficile* by reducing environmental contamination and transmission from contaminated hands. Finally, COVID-19 led to prolonged hospital admissions in patients at high risk for HO-CDI, including older patients and those with comorbidities. Given these opposing forces, we studied the association of COVID-19 admissions at our medical center on the incidence of admissions complicated with HO-CDI during the same month to better understand the complex risk factors for HO-CDI. This work is part of a larger study that aims to create a hospital geographic information system to identify and investigate clusters of infections. The objective of this study was to assess the relative risk of HO-CDI during each month of the COVID-19 pandemic (March 2020–May 1, 2021), relative to the historical expectation based on patient characteristics.

## Methods

### Patient population

In this retrospective cohort study, we included all patients who were admitted to The Ohio State University Wexner Medical Center (OSUWMC) for an inpatient hospital stay at one of our acute-care hospitals between January 2018 and May 2021 excluding prisoners, children, those who presented with *C. difficile* (test positive within first 4 days of hospitalization), and those with a length of stay (LOS) of <4 days. We excluded those with a <4-day LOS because they were not available to be diagnosed with HO-CDI because they left the hospital before day 4.

Data collected on these patients included race and ethnicity, sex, age, Charlson comorbidity score, antibiotic administration, admission and discharge dates, location stayed within the hospital, *C. difficile* test order time and result. Date of admission was used as the temporal index for a patient regardless of when in the admission the COVID-19 diagnosis or HO-CDI diagnosis was made.

Variables were chosen based on findings and conventions in prior literature and guidelines.^3,5^ In addition, we limited variables to those reliably captured in EHR data to aide in reproducibility. We used the following definitions of variables in the model: A COVID-19 admission was defined as one in which an ICD-10 code (U07.1) for COVID-19 was associated with the admission. An HO-CDI admission was defined as one in which the patient had a positive *C. difficile* polymerase chain reaction (PCR) on or after day 4 of hospitalization according to the National Healthcare Safety Network (NHSN) HO-CDI definition of a laboratory-confirmed *C. difficile* infection.^6^

For patients with HO-CDI, antibiotic use was defined as the total number of unique classes of antibiotics used 30 days prior to hospitalization and before a positive *C. difficile* test during hospitalization. For patients without HO-CDI, antibiotic use was defined as the total number of unique classes of antibiotics used 30 days prior to hospitalization and during hospitalization. We included 14 unique classes of antibiotic in this study: clindamycin, cephalosporins, carbapenems, fluoroquinolones, aztreonam, macrolides, penicillins, SMX-TMP, aminoglycosides, tetracyclines, vancomycin, oxazolidinones, daptomycin, and other (which included antibiotics not in previous classes). Oral vancomycin, fidaxomicin, metronidazole and topical antibiotics were not included in any of the classes. Antibiotics were categorized by an infectious disease subject-matter expert. For descriptive analysis, antibiotics were classified as high risk or low risk based on findings from prior literature.^7–9^ High-risk antibiotics included clindamycin, cephalosporins, carbapenems, fluoroquinolones, and piperacillin-tazobactam.

We split LOS into 2 categories: 4–10 days or ≥10 days. This division was for simplicity and was also based on prior literature conventions.^5^

Previous CDI was defined as a positive *C. difficile* result within a year prior to admission date of the current hospitalization. Total number of unique rooms was defined as the total number of unique patient rooms that each patient was transferred to during their hospitalization. This number did not include procedure rooms, only patient rooms. Building was defined as the hospital building from which a patient was discharged. Building was important to include because each building served different patient populations and thus had a different case mix and baseline risk for HO-CDI.

### Statistical methods

The main objective of this study was to explore temporal variation in monthly rates HO-CDI in the months prior to and the early months of the COVID-19 pandemic. Patient hospitalizations and HO-CDI were assigned to the month of patient admission for the primary analysis. In a sensitivity analysis (included in the Supplementary Material online), we assigned HO-CDI cases to the month when the laboratory test was ordered. We estimated the expected number of monthly *C. difficile* infections assuming no temporal change in the risk, then we used a Poisson regression model to estimate monthly standardized incidence ratios (SIR) to compare the observed number of infections to the expected number. This approach was similar to that used by the NHSN for monitoring healthcare-associated infections.^10^ Recognizing that the risk of *C. difficile* can vary by patient-level factors, we computed expected counts that adjusted for hospitalized patients’ characteristics to ensure fair comparisons over time because the characteristics of the hospitalized patient population may have changed over time, particularly during the COVID-19 pandemic.^10^

We assessed potential heterogeneity over time; therefore, the expected number of infections was computed assuming that the rates of infection are constant over time, conditional on a set of patient factors. This approach was similar to that of a null assumption that patient risk of HO-CDI did not depend on the month of hospitalization. First, we computed the monthly expected number of HO-CDI cases by aggregating the estimated patient probabilities of HO-CDI for all patients admitted in each month of the study. We used a logistic regression model to estimate the probability of HO-CDI for each patient in the study. The model included each patient’s Charlson score, age at admission, number of antibiotic classes used, race, an indicator of whether length of stay was greater than or equal to 10 days, whether they had a prior CDI diagnosis, the number of room transfers in the hospital stay, and an indicator of the hospital building. We included a sensitivity analysis in the supplement that uses cubic spline effects for all continuous covariates in the logistic regression model. Collinearity between variables was assessed using generalized variance-inflation factors (GVIFs). In terms of collinearity, GVIFs were <2 for all variables, and most had values close to 1, indicating lack of collinearity in our variables. Model discrimination was assessed by estimating area under the receiver operating curve (AUC), and calibration was assessed using the Hosmer-Lemeshow test. Using the estimated infection probability for each patient, the expected number of infections for the medical center was computed by summing the probabilities of all patients hospitalized during each month. The expected number of HO-CDI served to normalize for differences in the hospitalized patient populations over time, which may have been exacerbated by systemic changes during the initial phase of the COVID-19 pandemic.

To explore whether the number of observed infections differed from what was expected, a Poisson regression model was used for the observed counts including a temporal cubic spline, an autoregressive random effect at each time point to account for overdispersion and additional temporal correlation, and the log of the expected number of infections as an offset term. The model was fit within the Bayesian paradigm so prior distributions were required. We used noninformative but proper prior distributions for all parameters. The model was computed using a Markov chain Monte Carlo algorithm implemented in the nimble R package that was run for 500,000 iterations, discarding the first 250,000 and thinning by 50.^11^ The posterior distributions were summarized using the posterior mean and 90% equal-tail credible intervals (CIs). Full model details are included in the Supplementary Materials (online).

This study was approved by the university’s institutional review board. Patient data were delivered to the research team in a coded-limited format.

## Results

Table 1 shows the patient population included in our study. HO-CDI patients were slightly older, with a higher Charlson score and more likely to have a stay ≥10 days. A greater proportion of patients in the HO-CDI group received high-risk antibiotics and 2 or 3 antibiotic classes compared to the non–HO-CDI group. HO-CDI patients also had a longer average duration of high-risk antibiotic use. Supplementary Table S1 (online) shows how these factors varied over the study period.

**Table 1.** Patient Characteristics During the Study Period (HO-CDI vs Non-HO-CDI)

Variable	Admission Type, No. (%)^a^	
	HO-CDI (n = 712)	Non HO-CDI (n = 100,983)
**Race**		
White	579 (81.3)	74,783 (74.1)
Black	96 (13.5)	21,151 (20.9)
Other	37 (5.2)	5,049 (5.0)
Age, mean y	61.5	58.8
**Ethnicity**		
Not Hispanic	697 (97.9)	98,518 (97.6)
Hispanic	13 (1.8)	1,795 (1.8)
Unknown	2 (0.3)	670 (0.7)
**Sex**		
Female	353 (49.6)	49,138 (48.7)
Male	359 (50.4)	51,844 (51.3)
**Charlson score**	4.3	3.9
**Extend stay**		
4–9 d	112 (15.7)	69,056 (68.4)
10+ d	600 (84.3)	31,927 (31.6)
**Previously** ***C. difficile*** **positive**		
Yes	42 (5.9)	1,482 (1.5)
No	670 (94.1)	99,501 (98.5)
Total number of rooms transfer, mean	4.2	3.5
**No. of classes of antibiotics given**		
0	105 (14.7)	28,353 (28.1)
1	157 (22.1)	22,679 (22.5)
2	212 (29.8)	23,874 (23.6)
3	144 (20.2)	15330 (15.2)
4	68 (9.6)	7509 (7.4)
5+	26 (3.7)	3238 (3.2)
**Risk of antibiotic given**		
High-risk	557 (78.2)	63,553 (62.9)
Low-risk	50 (7.0)	9,074 (9.0)
No antibiotic	105 (14.7)	28,356 (28.1)
Days on high-risk antibiotic, mean	5.3	4.0
**Buildings**		
A	77 (10.8)	10,926 (10.8)
B	96 (13.5)	12,648 (12.5)
C	11 (1.5)	3,029 (3.0)
D	72 (10.1)	13,168 (13.0)
E	284 (39.9)	31,794 (31.5)
F	93 (13.1)	17,619 (17.4)
G	79 (11.1)	11,752 (11.6)

a Units unless otherwise specified.

Table 2 shows the final model used to adjust for patient characteristics and compute the expected number of HO-CDI cases. The AUC was 81.2%, showing excellent discrimination and no evidence of lack of fit (*P* = .98). This finding suggests that the logistic regression model was adequate for providing case-mix–adjusted, expected HO-CDI counts.

**Table 2.** Multivariable Model Estimating Probability of HO-CDI

Variable	Odds Ratio	95% CI	*P* Value
**Race**			
White	Ref		
Black	0.61	(0.49–0.77)	<.0001
Other	0.93	(0.65–1.29)	.68
Age	1.01	(1.003–1.01)	.002
Charlson score	0.98	(0.96–1.01)	.17
**Extended stay**			
<10 d	Ref		
10+ d	11.38	(9.25–14.12)	<.0001
**Previously** ***C. difficile*** **positive**			
Yes	4.38	(3.12–5.99)	<.0001
No	Ref		
Total room transfers, no.	1.09	(1.05–1.13)	<.0001
**Antibiotic use**			
0	Ref		
1	1.31	(1.02–1.69)	.03
2	1.27	(1.00–1.62)	.05
3	1.02	(0.79–1.33)	.88
4	0.86	(0.62–1.17)	.33
5+	0.59	(0.37–0.91)	.02
**Buildings**			
A	Ref		
B	1.11	(0.82–1.50)	.52
C	0.31	(0.15–0.57)	<.001
D	1.13	(0.81–1.57)	.48
E	1.38	(1.05–1.82)	.02
F	0.80	(0.59–1.09)	.15
G	0.85	(0.12–1.18)	.33

Note. CI, credible interval.

**Figure 1.**
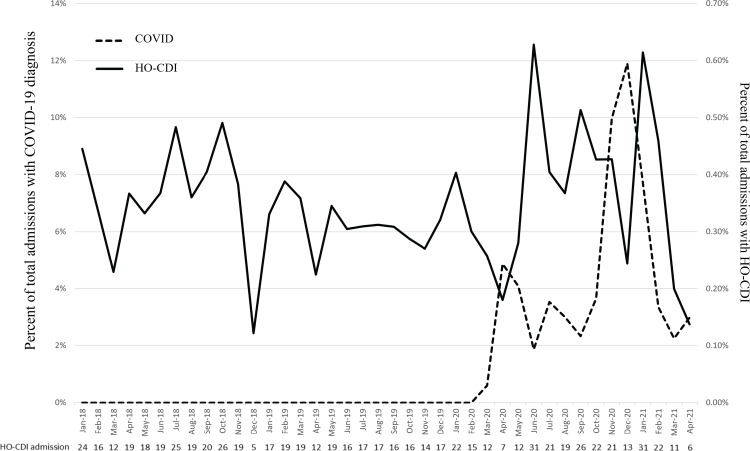
Percentage of total admissions each month that were associated with a COVID-19 diagnosis or hospital-onset Clostridioides difficile infection (HO-CDI).

Figure 1 shows the percentage of total admissions associated with COVID-19 or HO-CDI over time. It also shows the number of HO-CDI–related admissions per month in the table below Figure 1. Looking at the unadjusted data, when COVID-19 admissions were at their highest, HO-CDI–associated admissions were low. This finding held true for the surges in Spring 2020 and December 2020. Conversely, when COVID-19 admissions were a lower proportion of total admissions, we detected increased rates of admissions complicated by HO-CDI.

**Figure 2.**
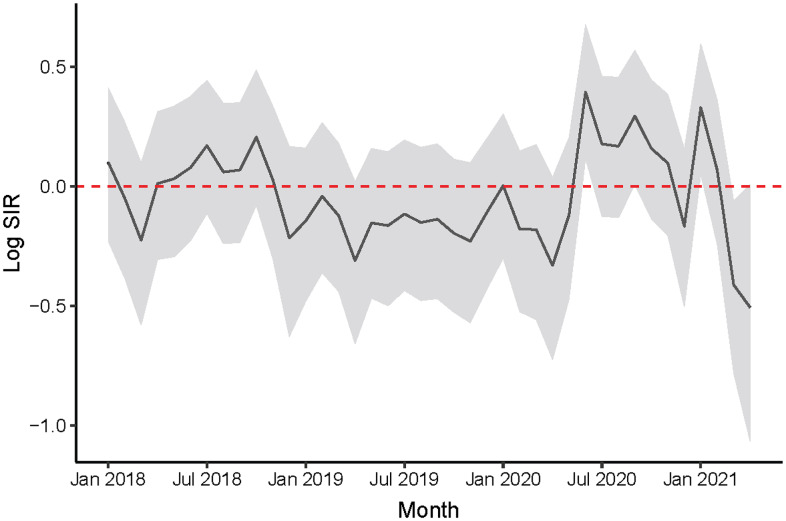
Posterior mean log standardized incidence ratio (SIR) and 90% credible interval for hospital-onset *Clostridioides difficile* infection (HO-CDI) by month from January 2018 to April 2021.

Figure 2 shows the posterior mean of the log SIR by month with a 90% CI. The dashed line at 0 reflects when the observed and expected number of HO-CDI are equal (ie, SIR, 1). For most months, the credible interval covers 0, indicating that after accounting for variability, the observed number of HO-CDI cases are compatible with what we expected. However, we noted several months during the pandemic when HO-CDI differed from what we expected. The posterior probability that HO-CDI incidence exceeded the expectation in June 2020 was 0.99 (SIR, 1.48; 90% CI, 1.12–1.97) and in January 2021 this probability was 0.98 (SIR, 1.39; 90% CI, 1.05–1.82). This finding suggests that rates of HO-CDI were 48% and 39% above expected during June 2020 and January 2021, respectively. The opposite was observed in March 2021, when the posterior probability that the observed HO-CDI was less than expected was 0.97 (SIR, 0.66; 90% CI, 0.46–0.94), suggesting a 34% reduction in rates relative to the expectation. As shown in the Supplementary Material (online), the results were similar when the expected counts were computed using splines in the logistic regression model and when the HO-CDI cases were assigned to the month the test was ordered instead of the month of patient admission (Supplementary Figs. S3 and S4 online).

## Discussion

In this study, there was no overall change in HO-CDI during the early COVID-19 pandemic; however, there were months when the observed number of cases of HO-CDI were higher or lower than we expected solely due to the case mix. This finding suggests that there may have been some impact of COVID-19 on HO-CDI that went beyond a change in patient characteristics. The COVID-19 pandemic was an unprecedented event that affected all aspects of daily life, including those within the healthcare system. For example, in the early pandemic there were reports of routine preventative visits being delayed or cancelled, PPE and staffing shortages, workforce burnout, and exacerbation of disparities in health outcomes and access to care.^12,13^ In addition, there were major changes to infection prevention guidance and standard practices (eg, universal masking). These effects and others may have contributed to our findings; however, they were difficult to quantify and track. We did assess whether changes in testing could have contributed. We examined the number of *C. difficile* tests sent after 4 days over this period. Fewer tests were ordered in early spring 2020, but this was a time of fewer admissions (Supplementary Fig. S2 online). Diagnostic testing for CDI at our health system is done using PCR and did not change over the study period.

Several other groups have assessed CDI incidence during the early COVID-19 pandemic.^14–16^ Allegretti et al^15^ performed a retrospective analysis of 9 hospitals in Massachusetts from February 2020 to November 2020 and found no evidence of an increased rate of CDI among COVID-19 patients. Ponce-Alonso et al^14^ looked at CDI incidence during a 2-month peak of the pandemic in Spain in early 2020 and found a lower rate of CDI compared to a historical control period. In a retrospective review of a tertiary-care center in New York, Luo et al^16^ did not find a significant difference between the prepandemic HO-CDI SIR and the HO-CDI SIR from February–June 2020. These researchers did note a trend toward higher-risk antibiotic exposures and increased LOS during the pandemic. Overall, these studies found no increase in CDI during the early pandemic despite increased use of antibiotics. Our study differed from these in that we included an additional year of pandemic data and we included additional individual patient-level risk factors. Instead of looking at the overall incidence of HO-CDI, we looked at the variation over the months of the pandemic. Notably, in contrast to the findings of Luo et al, we did not find a major difference in prolonged LOS between 2019 and 2020 (Supplementary Table 1 online). The mean number of unique antibiotic classes was generally stable over the years of the study, although a greater proportion of patients during 2020 received high-risk antibiotics. Also, patients with HO-CDI in 2020 and 2021 were less likely to have a history of CDI in the past, perhaps suggesting that other risk factors are contributing more than *C. difficile* colonization.

Interesting findings of our study included the low incidence of HO-CDI in the months of highest COVID-19 incidence and the high incidence of HO-CDI in the 1–2 months following COVID-19 spikes. The decreased incidence may have been due to increased use of PPE and hand hygiene during this time, as has been suggested in previous studies. Furthermore, the increased use of ultraviolet light as part of terminal cleaning procedure for all COVID-19 patient rooms could have affected the number of *C. difficile* cases during times of high COVID-19 admissions. The increase in HO-CDI when COVID-19 admissions were low could be related to the increased antibiotic use hospital-wide in the months prior. Some evidence indicates that antibiotic use in a patient increases the risk of CDI for a future patient in that same room.^17^ Additionally, this could be related to patients who had been admitted with severe COVID-19 but remained hospitalized and critically ill weeks to months later. These patients are very high risk for HO-CDI and could have led to environmental contamination and in-hospital transmission.

The logistic regression model was developed to adjust for case mix, not to identify or assess novel risk factors. In general, the model findings were in line with previous research. We included race in the model because there is a known disparity in CDI. Prior studies have shown that although HO-CDI incidence is higher in white patients, mortality and severe CDI are higher in Black patients.^18^ In our model, Black race was associated with a lower risk of HO-CDI when compared to white race, even after adjusting for other factors. Unintended consequences for the inclusion of race in the model should be assessed if this model is used for prospective risk assessment. The total number of room transfers was also associated with the outcome. This association was reported in a previous study by our group and could have been due to exposure to multiple hospital environments.^19^

Our study had several limitations. We used the date of admission as our temporal variable, yet HO-CDI could have occurred any time after day 4 of admission. On average, the HO-CDI cases occurred 11.86 days after admission. Our sensitivity analysis using order date instead of admission date showed similar findings although less pronounced. Given that expected cases were determined based on the characteristics of patients admitted per month, we favored counting the *C. difficile* cases in the same month in which the risks were counted. Given the study design, we were not able to show causation between COVID-19 surges and the variations in HO-CDI incidence. We only included data through the first year of the pandemic, so we were unable to determine whether the trend persisted. However, compared to the current literature, we had a more longitudinal view of the impact of COVID-19. There is some value to looking at only the first year because for the majority of the year, healthcare workers and patients had not been vaccinated and PPE use and behavior may have been different than at later stages in the pandemic. Finally, some patients with COVID-19 in the early pandemic were not included, and we excluded prisoners from our analysis. In Ohio, a significant portion of the first pandemic wave occurred in the incarcerated population.^20^

Our findings suggest that although overall incidence of HO-CDI did not increase during the first year of the COVID-19 pandemic, variations seemed to trend with COVID-19 incidence that cannot be fully explained by changing patient characteristics. Additional work should focus on whether this trend continued throughout the remaining pandemic and what factors may have contributed to reduced HO-CDI incidence when COVID-19 numbers were highest.

## Supplementary material

For supplementary material accompanying this paper visit https://doi.org/10.1017/ice.2023.171. click here to view supplementary material

## References

[1] COVID-19 impact on HAIs in 2020. Centers for Disease Control and Prevention website. https://www.cdc.gov/hai/data/portal/covid-impact-hai.html#anchor_1654807344682. Accessed September 13, 2023.

[2] Leffler DA , Lamont JT. *Clostridium difficile* infection. N Engl J Med 2015;372:1539–1548. 25875259 10.1056/NEJMra1403772

[3] McDonald LC , Gerding DN , Johnson S , et al. Clinical practice guidelines for *Clostridium difficile* infection in adults and children: 2017 update by the Infectious Diseases Society of America (IDSA) and Society for Healthcare Epidemiology of America (SHEA). Clin Infect Dis 2018;66:e1–e48. 29462280 10.1093/cid/cix1085 PMC6018983

[4] Rawson TM , Moore LSP , Zhu N , et al. Bacterial and fungal coinfection in individuals with coronavirus: a rapid review to support COVID-19 antimicrobial prescribing. Clin Infect Dis 2020;71:2459–2468. 32358954 10.1093/cid/ciaa530 PMC7197596

[5] Marley C , el Hahi Y, Ferreira G, Woods L, Ramirez Villaescusa A. Evaluation of a risk score to predict future *Clostridium difficile* disease using UK primary care and hospital data in Clinical Practice Research Datalink. Hum Vaccin Immunother 2019;15:2475–2481. 30945972 10.1080/21645515.2019.1589288 PMC6816380

[6] National Healthcare Safety Network. MDRO & CDI protocol, 2022. Centers for Disease Control and Prevention website. https://www.cdc.gov/nhsn/psc/cdiff/index.html. Accessed September 13, 2023.

[7] Webb BJ , Subramanian A , Lopansri B , et al. Antibiotic exposure and risk for hospital-associated *Clostridioides difficile* infection. Antimicrob Agents Chemother 2020;64:e02169–19. 31964789 10.1128/AAC.02169-19 PMC7179314

[8] Brown KA , Langford B , Schwartz KL , Diong C , Garber G , Daneman N. Antibiotic prescribing choices and their comparative *C. difficile* infection risks: a longitudinal case-cohort study. Clin Infect Dis 2021;72:836–844. 32069358 10.1093/cid/ciaa124 PMC7935390

[9] Tabak YP , Srinivasan A , Yu KC , et al. Hospital-level high-risk antibiotic use in relation to hospital-associated *Clostridioides difficile* infections: retrospective analysis of 2016–2017 data from US hospitals. Infect Control Hosp Epidemiol 2019;40:1229–1235. 31522695 10.1017/ice.2019.236 PMC9390868

[10] National Healthcare Safety Network. The NHSN standardized infection ratio (SIR) a guide to the SIR. Centers for Disease Control and Prevention website. https://www.cdc.gov/nhsn/pdfs/ps-analysis-resources/nhsn-sir-guide.pdf. Accessed September 13, 2023.

[11] de Valpine P , Turek D , Paciorek CJ , Anderson-Bergman C , Lang DT , Bodik R. Programming with models: writing statistical algorithms for general model structures with NIMBLE. J Comput Graph Stat 2017;26:403–413.

[12] Office of Inspector General. Hospitals reported that the COVID-19 pandemic has significantly strained healthcare delivery. US Department of Health and Human Services website. https://oig.hhs.gov/oei/reports/OEI-09-21-00140.asp. Published 2021. Accessed September 13, 2023.

[13] Office of the Assistant Secretary for Planning and Evaluation. Impact of the COVID-19 pandemic on the hospital and outpatient clinician workforce: challenges and policy responses (issue brief no. HP-2022-13). US Department of Health and Human Services website. https://aspe.hhs.gov/reports/covid-19-health-care-workforce. Published 2022. Accessed September 13, 2023.

[14] Ponce-Alonso M , Sáez De La Fuente J , Rincón-Carlavilla A , et al. Impact of the coronavirus disease 2019 (COVID-19) pandemic on nosocomial *Clostridioides difficile* infection. Infect Control Hosp Epidemiol 2021;42:406–410. 32895065 10.1017/ice.2020.454 PMC7520631

[15] Allegretti JR , Nije C , McClure E , et al. Prevalence and impact of *Clostridioides difficile* infection among hospitalized patients with coranavirus disease 2019. JGH Open 2021;5:622–625. 34013064 10.1002/jgh3.12497 PMC8114993

[16] Luo Y , Grinspan LT , Fu Y , et al. Hospital-onset *Clostridioides difficile* infections during the COVID-19 pandemic. Infect Control Hosp Epidemiol 2021;42:1165–1166. 32962772 10.1017/ice.2020.1223 PMC7545245

[17] Freedberg DE , Salmasian H , Cohen B , Abrams JA , Larson EL. Receipt of antibiotics in hospitalized patients and risk for *Clostridium difficile* infection in subsequent patients who occupy the same bed. JAMA Intern Med 2016;176:1801–1808. 27723860 10.1001/jamainternmed.2016.6193 PMC5138095

[18] Argamany JR , Delgado A , Reveles KR. *Clostridium difficile* infection health disparities by race among hospitalized adults in the United States, 2001 to 2010. BMC Infect Dis 2016;16:454. 27568176 10.1186/s12879-016-1788-4 PMC5002147

[19] McHaney-Lindstrom M , Hebert C , Flaherty J , Mangino JE , Moffatt-Bruce S , Dowling Root E. Analysis of intrahospital transfers and hospital-onset *Clostridium difficile* infection. J Hosp Infect 2019;102:168–169. 30172746 10.1016/j.jhin.2018.08.016 PMC7321831

[20] COVID-19 current month inmate testing. Ohio Department of Rehabilitation and Correction website. https://coronavirus.ohio.gov/static/reports/DRCCOVID-19Information.pdf. Accessed September 13, 2023.

