# Supplementary material for: Describing the monthly variability of hospital-onset Clostridioides difficile during early coronavirus disease 2019 (COVID-19) using electronic health record data
Source: Infect Control Hosp Epidemiol. 2023 Oct 9;45(3):329–34. doi: 10.1017/ice.2023.171 (PMC10933504; doi:10.1017/ice.2023.171)
Supplement: Supplementary file 1 [file S0899823X2300171Xsup001.pdf]

Supplemental Table S1.

Table S1. Patient characteristics from 2018 to 2021(HO-CDI vs. Non-HO-CDI)

	2018(n=30203)		2019(n=32184)		2020(n=29592)		2021(n=9716)	
**Variable**	Case (n=220)	Non-case (n=29983)	Case (n=199)	Non-case (n=31985)	Case (n=223)	Non-case (n=29369)	Case (n=70)	Non-case (n=9646)
**Race,** n (%)								
White	191 (86.8)	22352 (74.5)	152 (76.4)	23549 (73.6)	182 (81.6)	21647 (73.7)	54 (77.1)	7235 (75.0)
Black	20 (9.1)	6115 (20.4)	30 (15.1)	6839 (21.4)	32 (14.3)	6252 (21.3)	14 (20)	1945 (20.2)
Other	9 (4.1)	1516 (5.1)	17 (8.5)	1597 (5.0)	9 (4.0)	1470 (5.0)	2 (2.9)	466 (4.8)
**Age,** Mean	61.2	58.1	62.7	58.7	60.7	59.1	60.9	59.9
**Ethnicity,** n (%)								
Not Hispanic	220 (100)	29276 (97.6)	190 (95.5)	31248 (97.7)	219 (98.2)	28589 (97.3)	68 (97.1)	9405 (97.5)
Hispanic	0	505 (1.68)	7 (3.5)	543 (1.7)	4 (1.8)	557 (1.9)	2 (2.9)	190 (2.0)
Unknown	0	202 (0.7)	2 (1.0)	194 (0.6)	0	223 (0.8)	0	51 (0.5)
**Sex,** n (%)								
Female	112 (50.9)	14748 (49.2)	107 (53.8)	15648 (48.9)	101 (45.3)	14036 (47.8)	33 (47.1)	4706 (48.8)
Male	108 (49.1)	15235 (50.8)	92 (46.2)	16337 (51.1)	122 (54.7)	15332 (52.2)	37 (52.9)	4940 (51.2)
**Charlson score,** Mean	4.2	3.9	3.9	3.9	4.7	3.9	4.3	3.8
**Length of stay,** n (%)								
4-9 days	41 (18.6)	20734 (69.2)	27 (13.6)	21775 (68.1)	30 (13.5)	19936 (67.9)	14 (20.0)	6611 (68.5)
10+ days	179 (81.4)	9249 (30.8)	172 (86.4)	10210 (31.9)	193 (87.7)	9433 (32.1)	56 (80.0)	3035 (31.5)
**Previous *C.diff* positive,** n (%)								
Yes	18 (8.2)	470 (1.6)	14 (7.0)	409 (1.3)	8 (3.6)	446 (1.5)	2 (2.9)	157 (1.6)
No	202 (91.8)	29513 (98.4)	185 (93.0)	31576 (98.7)	215 (96.4)	28923 (98.5)	68 (97.1)	9489 (98.4)

**Total number of rooms transfer,** Mean	4.1	3.6	4.3	3.5	4.4	3.4	4.0	3.1
**Number of classes of antibiotic used,** n (%)								
0	35 (15.9)	8230 (27.4)	36 (18.1)	9366 (29.3)	25 (11.2)	7933 (27.0)	9 (12.9)	2824 (29.3)
1	51 (23.2)	6747 (22.5)	39 (19.6)	7232 (22.6)	48 (21.5)	6501 (22.1)	19 (27.1)	2199 (22.8)
2	64 (29.1)	6896 (23.0)	61 (30.7)	7369 (23.0)	64 (28.7)	7263 (24.7)	23 (32.9)	2346 (24.3)
3	40 (18.2)	4640 (15.5)	40 (20.1)	4642 (14.5)	51 (22.9)	4612 (15.7)	13 (18.6)	1436 (14.9)
4	23 (10.5)	2398 (8.0)	17 (8.5)	2349 (7.3)	22 (9.9)	2158 (7.3)	6 (8.6)	604 (6.3)
5+	7 (3.2)	1072 (3.6)	6 (3.0)	1027 (3.2)	13 (5.8)	902 (3.1)	0	237 (2.5)
**Risk of antibiotic used, n (%)**								
High	169 (76.8)	19036 (63.5)	148 (74.4)	19537 (61.1)	185 (83.0)	19000 (64.7)	55 (78.6)	5978 (62.0)
Low	16 (7.3)	2717 (9.1)	15 (7.5)	3078 (9.6)	13 (5.8)	2435 (8.3)	6 (8.6)	843 (8.7)
No antibiotic	35 (15.9)	8230 (27.4)	36 (18.1)	9370 (29.3)	25 (11.2)	7934 (27.0)	9 (12.9)	2825 (29.3)
**Days on high-risk antibiotic, Mean**	4.9	3.8	4.8	3.7	6.3	3.9	4.4	3.7
**Buildings,** n (%)								
A	26 (11.8)	3209 (10.7)	25 (12.6)	3408 (10.7)	19 (8.5)	3210 (10.9)	7 (10.0)	1099 (11.4)
B	23 (10.5)	3745 (12.5)	31 (15.6)	4037 (12.6)	34 (15.2)	3726 (12.7)	8 (11.4)	1140 (11.8)
C	4 (1.8)	962 (3.2)	0	929 (2.9)	7 (3.1)	884 (3.0)	0	254 (2.6)
D	23 (10.5)	3985 (13.3)	23 (11.6)	4069 (12.7)	16 (7.2)	3815 (13.0)	10 (14.3)	1299 (13.5)
E	101 (45.9)	9316 (31.1)	74 (37.2)	10382 (32.5)	83 (37.2)	9158 (31.2)	26 (37.1)	2938 (30.5)

F	18<br>(8.2)	5098<br>(17.0)	27<br>(13.6)	5606<br>(17.5)	36<br>(16.1)	5165<br>(17.6)	12<br>(17.1)	1750<br>(18.1)
G	25<br>(11.4)	3652<br>(12.2)	19<br>(9.5)	3541<br>(11.1)	28<br>(12.6)	3397<br>(11.6)	7<br>(10.0)	1162<br>(12.0)

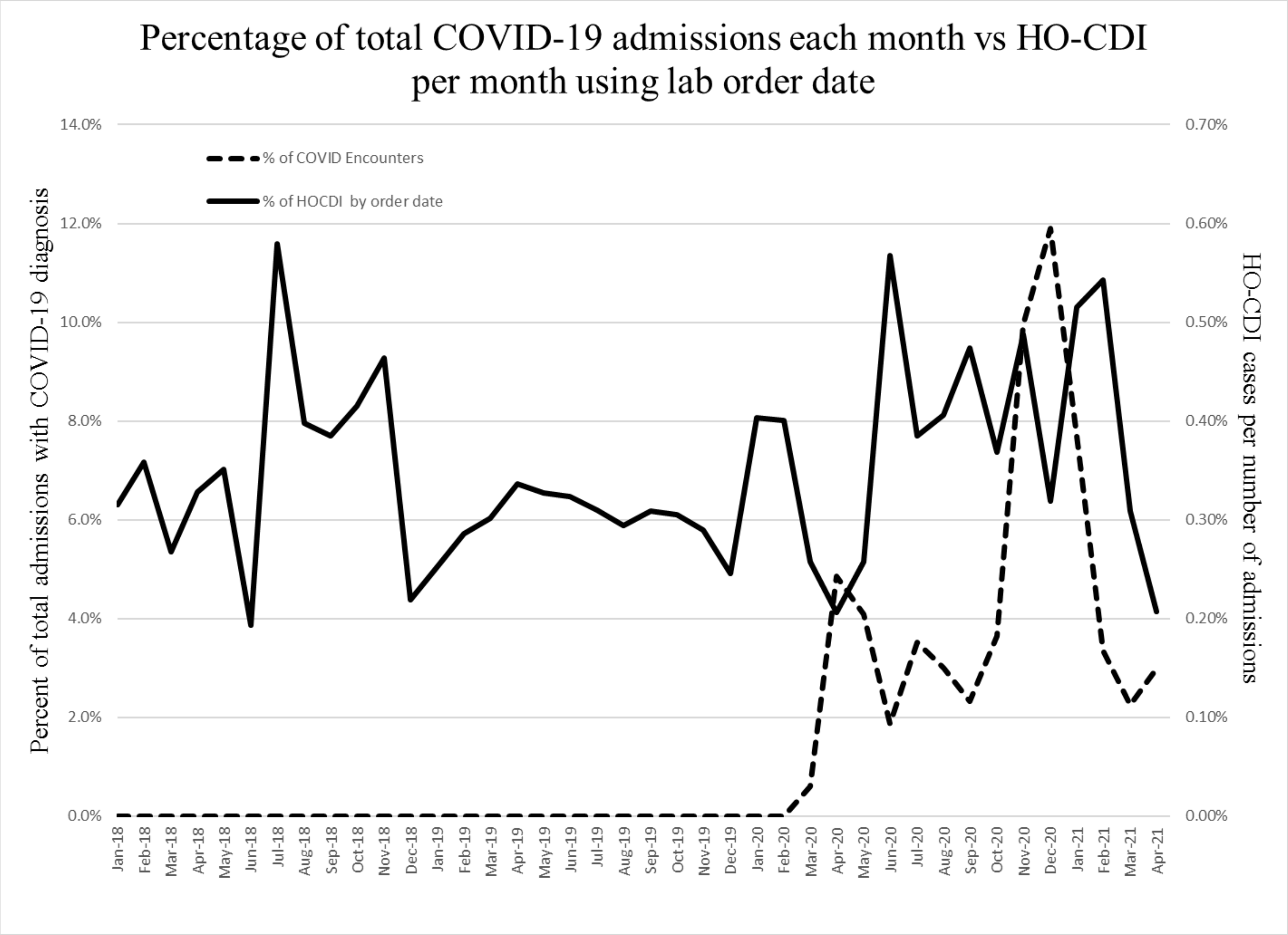

Percentage of total COVID-19 admissions each month vs HO-CDI per month using lab order date
% of COVID Encounters
% of HOCDI by order date
Percent of total admissions with COVID-19 diagnosis
HO-CDI cases per number of admissions
14.0%
12.0%
10.0%
8.0%
6.0%
4.0%
2.0%
0.0%
0.70%
0.60%
0.50%
0.40%
0.30%
0.20%
0.10%
0.00%
Jan-18
Feb-18
Mar-18
Apr-18
May-18
Jun-18
Jul-18
Aug-18
Sep-18
Oct-18
Nov-18
Dec-18
Jan-19
Feb-19
Mar-19
Apr-19
May-19
Jun-19
Jul-19
Aug-19
Sep-19
Oct-19
Nov-19
Dec-19
Jan-20
Feb-20
Mar-20
Apr-20
May-20
Jun-20
Jul-20
Aug-20
Sep-20
Oct-20
Nov-20
Dec-20
Jan-21
Feb-21
Mar-21
Apr-21

Figure S 1

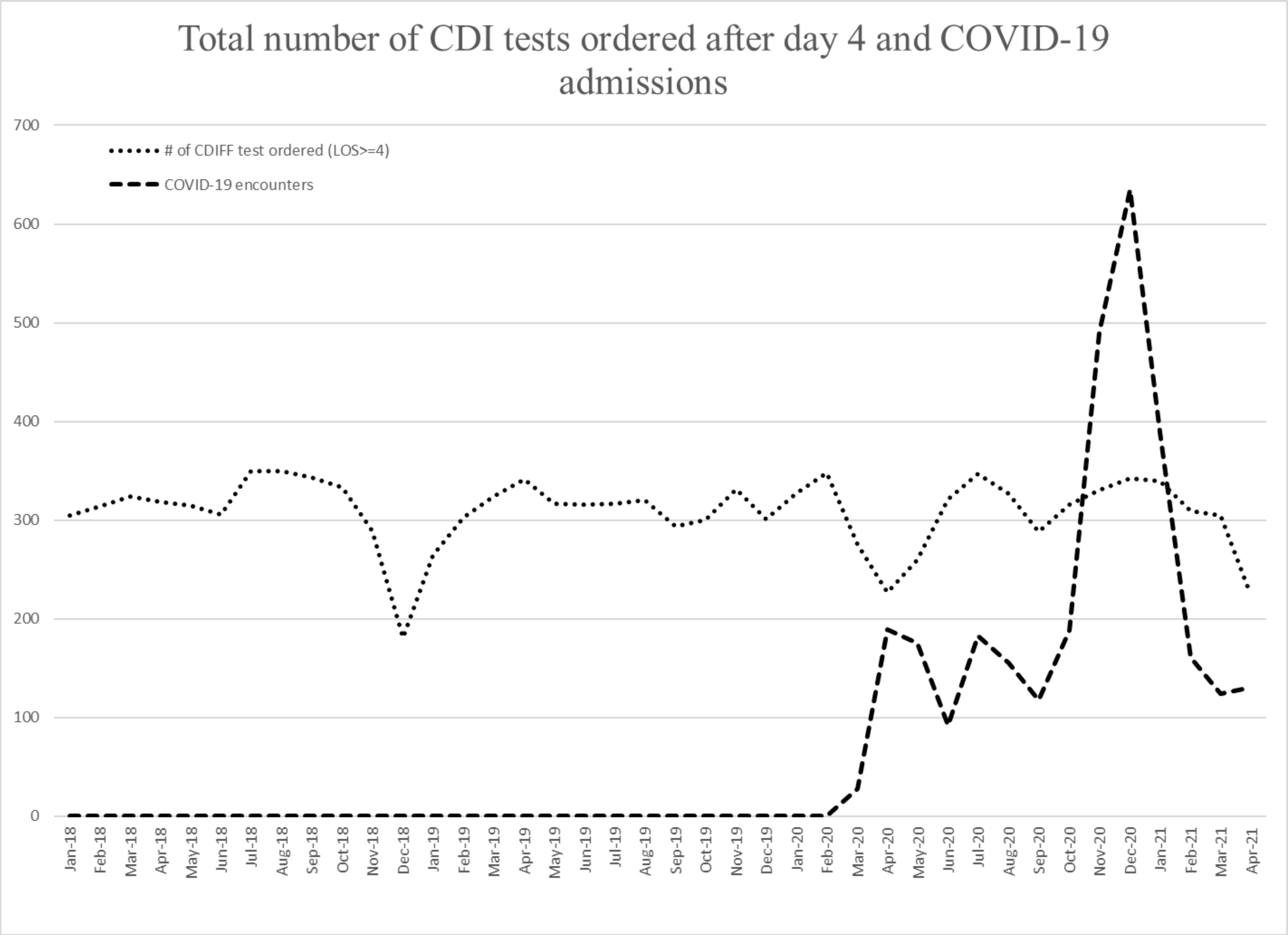

Total number of CDI tests ordered after day 4 and COVID-19 admissions
# of CDIFF test ordered (LOS>=4)
COVID-19 encounters
700
600
500
400
300
200
100
0
Jan-18
Feb-18
Mar-18
Apr-18
May-18
Jun-18
Jul-18
Aug-18
Sep-18
Oct-18
Nov-18
Dec-18
Jan-19
Feb-19
Mar-19
Apr-19
May-19
Jun-19
Jul-19
Aug-19
Sep-19
Oct-19
Nov-19
Dec-19
Jan-20
Feb-20
Mar-20
Apr-20
May-20
Jun-20
Jul-20
Aug-20
Sep-20
Oct-20
Nov-20
Dec-20
Jan-21
Feb-21
Mar-21
Apr-21

Figure S 2

Statistical Details

As described in the Methods section of the main paper, the main goal of the analysis is to compare the observed number of monthly HO-CDI with the expected number from January 2018 to May 2021. To do so, we use a standard Bayesian Poisson regression model to estimate the SIRs and their uncertainty and to assess trends over time. We assume the following data model:

$$Y_i \overset{ind}{\sim} \text{Poisson}(E_i \lambda_i)$$

where Y_i is the observed count of HO-CDI for month i, E_i is the expected count of HO-CDI for month i, and λ_i is the SIR for month i.

To compute E_i, we assume the probability of HO-CDI for a patient is constant over the study time period (i.e., no temporal heterogeneity). This implies that the SIR is comparing each month to the average over the time period. Thus, $E_i = \sum_{j=1}^{n_i} p_{j[i]}$ where n_i is the number of patients admitted in month i and $p_{j[i]}$ is the probability of HO-CDI for patient j admitted in month i. For the full Bayesian model, E_i is assumed to be known, as is typical for these models (Cressie and Wikle, 2011). We estimate p_j using the following logistic regression model:

$$\begin{aligned} Z_j &\overset{ind}{\sim} \text{Bernoulli}(p_j) \\ \text{logit}(p_j) &= \mathbf{X}_j' \boldsymbol{\beta} \end{aligned}$$

where Z_j is an indicator of whether patient j had HO-CDI, $\mathbf{X}_j$ is a vector of the covariates as described in the main text, and $\boldsymbol{\beta}$ is a vector of fixed effects. Through use of a logistic regression to compute p_j and thus E_i, we effectively adjust the expected count of HO-CDI for differences in the characteristics of the hospitalized patient population over time, which is particularly important during 2020 as hospitalization patterns were altered by the COVID-19 pandemic. In other words, assuming that patient risk of HO-CDI does not change over time given the covariates in $\mathbf{X}$, we are able to compute the expected count of HO-CDI cases for the set of patients actually hospitalized during any given month in a way that enables a fair comparison over time.

To model λ_i, we assume the following generalized linear model:

$$\log(\lambda_i) = \alpha_i + \epsilon_i$$

where α_i is the contribution at month i from a penalized cubic spline and ϵ_i is a random effect to account for overdispersion and temporal autocorrelation. The cubic spline was defined using the `jagam` function in the `mgcv` package in `R` (Wood, 2016). The spline is defined by $\alpha_i = \mu + \mathbf{B}_i \boldsymbol{\kappa}$ where μ is the intercept, $\mathbf{B}_i$ is vector of basis functions evaluated at time i, and $\boldsymbol{\kappa}$ is the vector of coefficients. We apply the penalty by assuming $\boldsymbol{\kappa} \sim N(\mathbf{0}, \tau \mathbf{S}^{-1})$ where $\mathbf{S}$ is a non-diagonal matrix multiplied by the smoothing parameter τ such that smaller values of τ result in a smoother function. To capture additional variation and temporal autocorrelation, we assume an autoregressive of order 1 structure for ϵ_i. That is, we assume

$$\begin{cases} \epsilon_i \sim N(0, \sigma^2) & i = 1 \\ \epsilon_i \sim N(\rho \epsilon_{i-1}, \sigma^2) & i > 1 \end{cases}$$

where σ^2 is a variance and ρ is an autoregressive parameter.

Since we fit the model within the Bayesian paradigm, we must specify prior distributions on all unknown parameters. We assume $\mu \sim N(0, 100)$ and $\rho \sim \text{Uniform}(-1, 1)$. For σ^2 and τ, we assume independent inverse gamma distributions with shape and scale equal to 0.5.

References

Cressie, N. and Wikle, C. K. (2011). *Statistics for Spatio-Temporal Data*. John Wiley & Sons.

Wood, S. N. (2016). Just Another Gibbs Additive Modeler: Interfacing JAGS and mgcv. *Journal of Statistical Software*, 75:1–15.

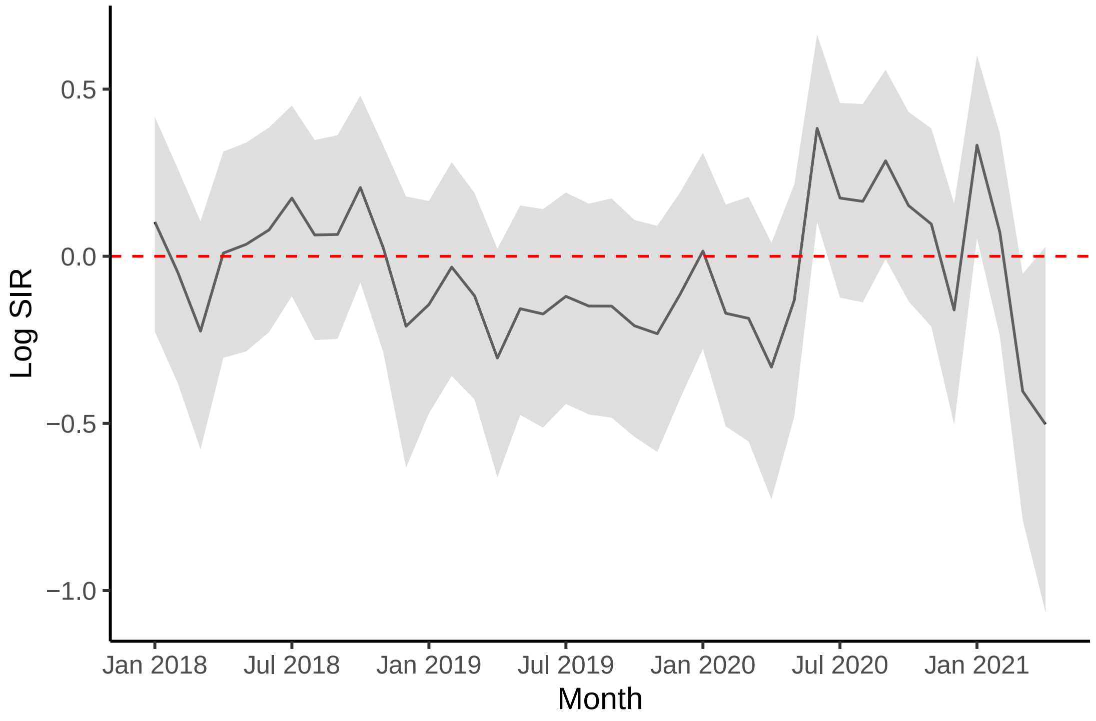

Figure S3: Posterior mean estimate of the log SIR by month with the associated 90% credible interval. Ex-pected case counts were computed using a logistic regression with cubic spline effects for all continuous covariates.

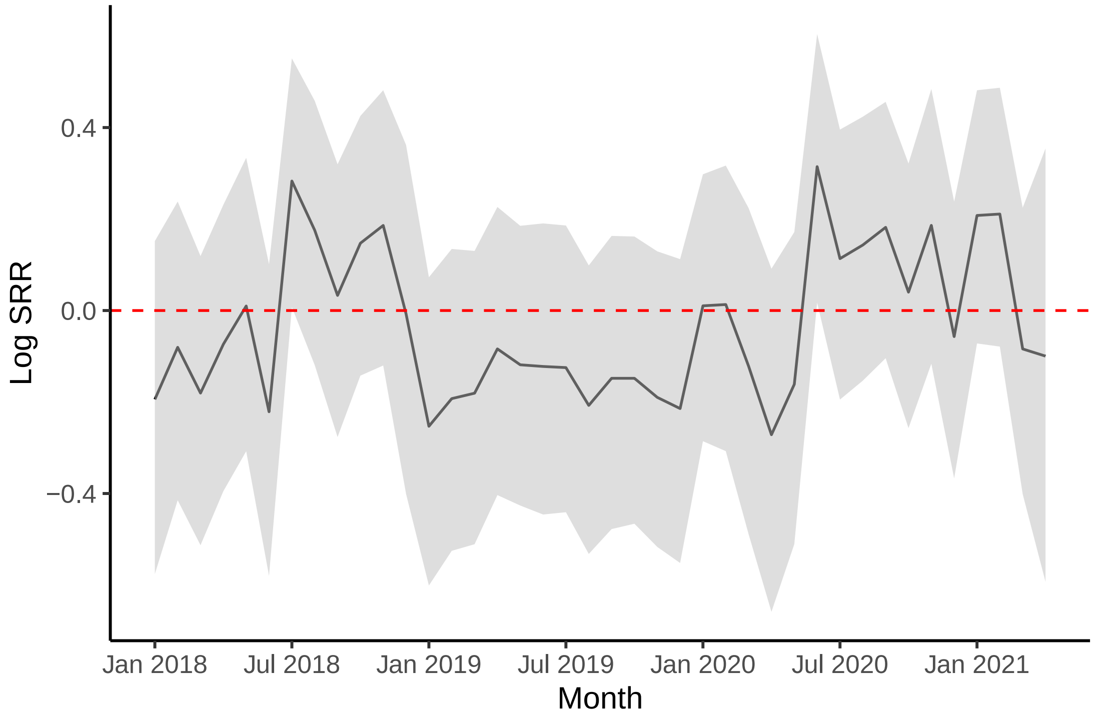

Figure S4: Posterior mean estimate of the log SIR by month with the associated 90% credible interval. HO-CDI infections are indexed to the date the lab test was ordered.
